# Medical Societies and Representation

**Published:** 1874-10-15

**Authors:** 


### Editorial Department.

### MEDICAL SOCIETIES AND REPRESENTATION.

THAT the organization of medical societies by which members of the profession are brought frequently in contact with each other for mutual improvement, has proved of very great value in promoting investigations, diffusing professional knowlege, and facilitating intercourse with, and respect for each other, no thoughtful man can doubt.

That such social organizations are capable of being made very much more useful in all these respects than they have hitherto been, is equally evident to all who have given the subject their attention. One important step in the work of increasing their usefulness, is to make them more truly representative of the whole profession and more ready of communication with each other. If the members of the profession in each city, county, or district would form themselves into a society for careful investigation of all matters of interest in their localities; if the basis of each state society was formed by delegates from the local societies, and the national organization by delegates from the state societies and such local ones as were in connection with them, the whole would not only form a complete bond of union, infusing more or less of the spirit and knowledge of each part throughout the whole, but also forming a medium of communication by which well matured plans of observation and investigation can be made uniform over large areas, and continuous over periods of time sufficient to obtain more complete and accurate results than are possible from isolated or individual enterprise.

It was to aid in thus perfecting the organization and usefulness of medical societies throughout the whole country, that certain amendments to the constitution of the American Medical Association were adopted last year at Detroit. These amendments restricted the appointment of delegates to that body, to the State and National Medical Societies, and such local and district societies as were recognized by representation in their respective state societies. The object was not merely to cut off the practice of receiving delegates from irregular and merely nominal medical institutions and societies, but to positively encourage the closer affiliation of the local societies in each state with their own state society. We hope the amendments themselves and the real objects of their adoption will be fully understood and appreciated by the profession in time to make their appointments to the next meeting of the National Association.

